# Clinical, imaging, and blood biomarkers to assess 1-year progression risk in fibrotic interstitial lung diseases—Development and validation of the honeycombing, traction bronchiectasis, and monocyte (HTM)-score

**DOI:** 10.3389/fmed.2022.1043720

**Published:** 2022-11-16

**Authors:** Guangyu Shao, Patricia Hawle, Kaveh Akbari, Andreas Horner, Rainer Hintenberger, Bernhard Kaiser, Bernd Lamprecht, David Lang

**Affiliations:** ^1^Department of Internal Medicine 4 – Pneumology, Kepler University Hospital, Linz, Austria; ^2^Medical Faculty, Johannes Kepler University Linz, Linz, Austria; ^3^Central Radiology Institute, Kepler University Hospital, Linz, Austria; ^4^Department of Internal Medicine 2, Kepler University Hospital, Linz, Austria

**Keywords:** traction bronchiectasis, honeycombing, monocyte count, forced vital capacity (FVC), diffusion capacity (DL), idiopathic pulmonary fibrosis, autoimmune disease, lung fibrosis

## Abstract

**Introduction:**

Progression of fibrotic interstitial lung disease (ILD) leads to irreversible loss of lung function and increased mortality. Based on an institutional ILD registry, we aimed to evaluate biomarkers derived from baseline patient characteristics, computed tomography (CT), and peripheral blood for prognosis of disease progression in fibrotic ILD patients.

**Methods:**

Of 209 subsequent ILD-board patients enregistered, 142 had complete follow-up information and were classified fibrotic ILD as defined by presence of reticulation or honeycombing using a standardized semi-quantitative CT evaluation, adding up typical ILD findings in 0–6 defined lung fields. Progression at 1 year was defined as relative loss of ≥10% in forced vital capacity, of ≥15% in diffusion capacity for carbon monoxide, death, or lung transplant. Two-thirds of the patients were randomly assigned to a derivation cohort evaluated for the impact of age, sex, baseline lung function, CT finding scores, and blood biomarkers on disease progression. Significant variables were included into a regression model, its results were used to derive a progression-risk score which was then applied to the validation cohort.

**Results:**

In the derivation cohort, age, monocyte count ≥0.65 G/L, honeycombing and traction bronchiectasis extent had significant impact. Multivariate analyses revealed the variables monocyte count ≥0.65 G/L (1 point) and combined honeycombing or traction bronchiectasis score [0 vs. 1–4 (1 point) vs. 5–6 lung fields (2 points)] as significant, so these were used for score development. In the derivation cohort, resulting scores of 0, 1, 2, and 3 accounted for 1-year progression rates of 20, 25, 46.9, and 88.9%, respectively. Similarly, in the validation cohort, progression at 1 year occurred in 0, 23.8, 53.9, and 62.5%, respectively. A score ≥2 showed 70.6% sensitivity and 67.9% specificity, receiver operating characteristic analysis for the scoring model had an area under the curve of 71.7%.

**Conclusion:**

The extent of honeycombing and traction bronchiectasis, as well as elevated blood monocyte count predicted progression within 1 year in fibrotic ILD patients.

## Introduction

Until recently, interstitial lung diseases (ILD) with an assumed underlying pathophysiological mechanism of inflammation, like hypersensitivity pneumonitis (HP) or ILD associated with autoimmune diseases, were mostly treated using anti-inflammatory therapies, e.g., immunomodulatory, or immunosuppressive agents (1). With few exceptions, (2–5) this was, however based on only little high-quality evidence. After the advent of the anti-fibrotic drugs Pirfenidone and Nintedanib had fundamentally changed the therapeutic landscape in IPF (6, 7), increasing evidence also suggested their use in systemic sclerosis (SSC)-ILD or progressive fibrosing ILD other than IPF (8–12). With regards to these advances, recent studies and guidelines support a treatment strategy based on disease phenotype, irrespective of the underlying ILD diagnosis (13). Patients with “inflammatory” ILD considered likely to respond to anti-inflammatory therapies should receive such treatment, however if progressive fibrosis occurs, anti-fibrotic agents should be used either as monotherapy or as an add-on (8, 13–17). However, in non-IPF ILD with fibrotic features in imaging that have not yet shown progression, existing evidence still does not allow to draw conclusions on which kind of treatment to be initiated primarily (18).

Numerous biomarkers have been reported to be associated with mortality and disease progression in IPF and other fibrotic ILD, such as the presence of honeycombing or traction bronchiectasis (19–21), disease extent (21, 22), previous functional worsening (23), peripheral blood monocyte count (24), or family history of ILD (25). High hopes also rest upon proteomic biomarker panels derived from patient blood, but those are not widely available in clinical practice yet (26). Some of these biomarkers have already been included into clinical scores, such as the gender-age-physiology (GAP) model for IPF and other ILD subtypes (27, 28), or the staging system by Goh et al. for SSC-ILD (22). However, particularly in the heterogeneous group of fibrotic non-IPF ILD, a risk prediction score offering guidance for initial clinical management has not been established yet.

We thus aimed to develop a scoring system for estimating 1-year progression-risk in a cohort of patients with radiologically evident fibrotic ILD based on our institutional ILD registry.

## Materials and methods

Patients evaluated in this study were retrospectively extracted from the institutional ILD registry of Johannes Kepler University Hospital Linz, which was conducted in concordance with the Declaration of Helsinki and was approved and reassessed on a yearly basis by the ethics committee of the Medical Faculty of Linz (study number I-26-17). This study was performed according to the Strengthening the Reporting of Observational Studies in Epidemiology (STROBE) guidelines for reporting observational studies (29).

As described in previous publications (30, 31), all patients discussed by the local ILD-board were included into a prospective registry between 2017 and 2021. Patients enregistered had undergone standardized baseline evaluation including high-resolution computed tomography (HRCT), blood analyses including autoimmune antibody screening, and pulmonary functions tests (PFT). To be included in the present analysis, patients were required to have fibrotic ILD as determined by the presence of reticular lung abnormalities or honeycombing on initial HRCT. Also, survival and PFT follow-up for at least 1 year after primary evaluation needed to be available. Anti-inflammatory or anti-fibrotic treatment was considered relevant and ILD-specific, when it had been given for a minimum of 6 weeks and when it was primarily prescribed due to ILD, but not for controlling other diseases or underlying conditions like extrapulmonary manifestations of rheumatoid arthritis.

High-resolution computed tomography images were acquired according to protocols suggested by the relevant guidelines(32). If clinically feasible, prone imaging was preferred to differ opacities in dependent lung areas from true interstitial lung abnormalities (33). During the respective ILD-board session, a specialist ILD-radiologist assessed the presence of parenchymal nodules, reticular abnormalities, honeycombing, consolidations, ground glass opacities, emphysema, mosaic attenuation, and traction bronchi(-ol)ectasis in an upper-, middle- and lower-lung area as defined by thirds of the largest cranio-caudal diameter in the sagittal reconstructions, leading to scores from zero to six, as described for our previously reported evaluations (30, 31). Each finding was then scored as absent, limited or abundant using cut-off values based on statistical modeling of the leading variables as explicated below. Additionally, aortic- and pulmonary artery diameters were measured and the number of lobes with visual signs of volume reduction was assessed.

Blood samples were analyzed using a Sysmex^®^ XN-3000 hematology analyzer (Sysmex Europe GmbH, Norderstedt, Germany) for blood cell counts and a Cobas^®^ 8,000 modular analyzer (Roche Diagnostics International AG, Rotkreuz, Switzerland) for C-reactive protein (CRP), lactate dehydrogenase (LDH), and rheumatoid factor. Autoimmune serology testing was performed *via* a EuroPatternMicroscope^®^, a Dynex^®^, and a EuroBlotOne^®^ platform by Euroimmun (EUROIMMUN Medizinische Labordiagnostika AG, Lübeck, Germany) for anti-nuclear (ANA), anti-neutrophil cytoplasmatic (ANCA) and other disease-specific antibodies, using the respective kits acquired from Euroimmun. Patients were considered to have significant autoimmune findings, if these fulfilled the serological domain of the interstitial pneumonia with autoimmune features (IPAF) criteria (34).

Pulmonary function tests included spirometry, body plethysmography, and measurement of diffusion capacity (JAEGER MasterScreen PFT/Body/Diffusion^®^, CareFusion, San Diego, United States of America), PFT biomarkers parameters specifically analyzed in this study were forced vital capacity (FVC, L/% predicted), forced expiratory volume in 1 s (FEV1, % predicted), FEV1/FVC ratio and diffusion capacity for carbon monoxide (DLCO, single breath method, mmol/(min × kPa)/%predicted). Normal values for spirometry were based on the GLI-2012 equations (35), those for body plethysmography and diffusion capacity on the 1993 ERS/ECCS regressions (36).

Progression of ILD at 1 year was defined as a composite endpoint of either ≥10% relative decrease in FVC, ≥15% in DLCO, by death or lung transplant within the first year after primary evaluation and ILD-board discussion, regardless of when the event had occurred within that time span. In patients who did not have follow-up lung function testing at 12 months but at least once after inclusion in the previous and in the subsequent year, the respective 12-months FVC and DLCO value was interpolated assuming a linear change.

Two-thirds of the eligible patients were randomly assigned to a derivation cohort used for score development: Baseline patient characteristics including PFT results, laboratory biomarkers and HRCT scores were evaluated for their properties to differ between progressive and non-progressive patients using a *t*-test, Mann–Whitney U test, Chi-Square-test or Fisher’s exact test depending on normal distribution and scales of measure. Biomarkers showing a clinically relevant signal in visual analysis and in statistical testing were further evaluated in a binary logistic regression model. If necessary, cut-off values for key prognostic variables were calculated using the CUTPOINTR-package in R (R: A language and Environment for Statistical Computing. R Foundation for Statistical Computing, Vienna, Austria; Version 3.6.0)^1^, using a manually defined level of significance (*p* < 0.05), the minimum number of patients per subgroup (>10% of total *n*) and the minimum number of cut-off points (≤2) to evaluate the optimum cut-off value by regression analysis. Odds ratios for variables found to have a significant interaction with disease progression were then used to create a weighed progression-risk score with an optimum AUC in the receiver operating characteristics (ROC) analyses. The resulting score was finally tested in the remaining third of patients as validation cohort. All statistical analyses were performed using R, for all tests performed, a *p*-value < 0.05 was regarded statistically significant.

## Results

Of a total of 209 patients enrolled between 2017 and 2021, 142 met the criteria to be included into the analysis. Most patients had been diagnosed with autoimmune-associated ILD (24%), followed by idiopathic NSIP (21%), and IPF (16%) as shown in Supplementary Table 1.

Respective baseline characteristics, PFT and HRCT findings for all patients, the derivation and the validation cohort are shown in Tables 1–3. There were no significant differences between the derivation and validation cohort except for the distribution of ground glass opacity extent. In the derivation cohort, a significant association with disease progression could be detected for older age (*p* = 0.021), absolute monocyte count (*p* = 0.001), honeycombing (*p* = 0.035), and traction bronchiectasis (*p* = 0.043).

**TABLE 1:** Baseline patient, treatment, and pulmonary function test characteristics in all patients, the derivation, and the validation cohort as well as in the derivation cohort according to progression at 1 year.

	All patients (*n* = 142)				Derivation cohort (*n* = 95)		
Variable	All patients (*n* = 142)	Derivation cohort (*n* = 95)	Validation cohort (*n* = 47)	*P*-value	Stable at 1 year (*n* = 59)	Progression at 1 year (*n* = 36)	*P*-value
**Baseline characteristics**							
Mean age (SE)	67.0 (1.1)	66.8 (1.3)	67.4 (1.8)	0.829	64.3 (1.8)	70.9 (1.5)	0.021
Age ≥ 70 years (%)	47.2	48.4	44.7	0.674	40.7	61.1	0.053
Female sex (%)	36.6	39.0	31.9	0.413	42.4	33.3	0.381
**Treatment characteristics** (%)							
Anti-inflammatory	52.1	57.9	40.4	0.183	64.4	47.2	0.310
Anti-fibrotic	12.0	9.5	17.0		6.8	13.9	
Anti-inflammatory and anti-fibrotic	7.0	5.3	10.6		3.4	8.3	
No ILD-specific therapy	28.9	27.3	31.9		25.4	30.6	
**Pulmonary functions tests; mean (SE)**							
FVC (L)	2.9 (0.1)	2.9 (0.2)	3.0 (0.2)	0.945	2.9 (0.1)	2.9 (0.1)	0.890
FVC (% pred.)	81.3 (1.5)	80.4 (2.0)	83.2 (2.3)	0.542	79.9 (2.7)	81.3 (3.1)	0.779
FEV1 (L)	2.3 (0.1)	2.4 (0.1)	2.2 (0.1)	0.306	2.3 (0.1)	2.3 (0.1)	0.869
FEV1 (% pred.)	82.8 (1.6)	82.4 (2.6)	84.0 (2.2)	0.732	80.5 (2.8)	85.4 (3.0)	0.279
FEV1/FVC	80.5 (0.7)	80.8 (0.9)	79.7 (1.0)	0.169	80.4 (1.3)	81.5 (1.2)	0.808
DLCO [mmol/(min × kPa)]	4.5 (0.1)	4.5 (0.2)	4.6 (0.2)	0.895	4.5 (0.2)	4.4 (0.2)	0.982
DLCO (% pred.)	55.2 (1.5)	54.7 (1.8)	56.2 (2.6)	0.593	54.6 (2.3)	54.8 (2.7)	0.730

*P*-values are for comparison between the respective groups. SE, standard error; ILD, interstitial lung disease; FVC, forced vital capacity; FEV1, forced expiratory volume in 1 s; DLCO, diffusion capacity for carbon monoxide. Bold values indicate statistically significant variables.

**TABLE 2:** Baseline peripheral blood biomarkers in all patients, the derivation, and the validation cohort as well as in the derivation cohort according to progression at 1 year.

	All patients (*n* = 142)				Derivation cohort (*n* = 95)		
Peripheral blood biomarkers [mean (SE)]	All patients (*n* = 142)	Derivation cohort (*n* = 95)	Validation cohort (*n* = 47)	*P*-value	Stable at 1 year (*n* = 59)	Progression at 1 year (*n* = 36)	*P*-value
Absolute leukocyte count (G/L)	8.8 (0.3)	8.7 (0.3)	9.0 (0.5)	0.447	8.7 (0.5)	8.6 (0.5)	0.517
Absolute neutrophil count (G/L)	6.3 (0.3)	6.3 (0.4)	6.2 (0.4)	0.544	6.5 (0.5)	6.0 (0.4)	0.833
Absolute lymphocyte count (G/L)	1.7 (0.1)	1.6 (0.1)	1.8 (0.1)	0.187	1.6 (0.1)	1.6 (0.1)	0.945
Absolute monocyte count (G/L)	0.6 (0.1)	0.6 (0.1)	0.7 (0.1)	0.144	0.5 (0.1)	0.7 (0.1)	**0.001**
Absolute eosinophil count (G/L)	0.2 (0.1)	0.2 (0.1)	0.2 (0.1)	0.685	0.2 (0.1)	0.2 (0.1)	0.638
C-reactive protein (mg/dL)	1.2 (0.2)	1.2 (0.2)	1.3 (0.5)	0.322	1.0 (0.2)	1.4 (0.4)	0.398
Lactate dehydrogenase (U/L)	248.3 (7.5)	251.7 (8.8)	241.3 (14.0)	0.299	256.3 (13.4)	244.1 (12.1)	0.903
Serological IPAF domain (%)	46.8	50.0	40.0	0.267	50.9	48.6	0.831

*P*-values are for comparison between the respective groups. SE, standard error; IPAF, interstitial pneumonia with autoimmune features. Bold values indicate statistically significant variables.

**TABLE 3:** Baseline computed tomography scores in all patients, the derivation, and the validation cohort as well as in the derivation cohort according to progression at 1 year.

		All patients (*n* = 142)				Derivation cohort (*n* = 95)		
Computed tomography finding scores (%)	Score	All patients (*n* = 142)	Derivation cohort (*n* = 95)	Validation cohort (*n* = 47)	*P*-value	Stable at 1 year (*n* = 59)	Progression at 1 year (*n* = 36)	*P*-value
Parenchymal nodules	0	79.6	75.8	87.2	0.139	69.5	86.1	0.118
	1–4	14.8	19.0	6.4		25.4	8.3	
	5–6	5.6	5.2	6.4		5.1	5.6	
Reticular abnormalities	0	1.4	1.1	2.1	0.494	0.0	2.8	0.071
	1–4	31.7	34.7	25.5		42.4	22.2	
	5–6	66.9	64.2	72.4		57.6	75.0	
Honeycombing	0	83.1	86.3	76.6	0.288	93.2	75.0	**0.035**
	1–4	11.3	8.4	17.0		5.1	13.9	
	5–6	5.6	5.3	6.4		1.7	11.1	
Ground glass opacities	0	56.3	53.7	61.7	**0.021**	49.2	61.1	0.366
	1–4	24.7	21.0	31.9		25.4	13.9	
	5–6	19.0	25.3	6.4		25.4	25.0	
Consolidations	0	78.2	77.9	78.7	0.917	76.3	80.6	0.389
	1–4	18.3	19.0	17.0		18.6	19.4	
	5–6	3.5	3.1	4.3		5.1	0.0	
Mosaic attenuation	0	80.3	76.8	87.2	0.143	74.6	80.6	0.619
	1–4	19.7	23.2	12.8		25.4	19.4	
	5–6	0.0	0.0	0.0		0.0	0.0	
Emphysema	0	80.3	79.0	83.0	0.360	83.1	72.2	0.284
	1–4	16.9	16.8	17.0		11.9	25.0	
	5–6	2.8	4.2	0.0		5.0	2.8	
Traction bronchiectasis	0	16.2	15.8	17.0	0.915	20.3	8.7	**0.043**
	1–4	64.1	65.2	61.7		67.8	61.1	
	5–6	19.7	19.0	21.3		11.9	30.6	
Pulmonary artery/aorta	0	87.3	86.3	89.4	0.608	89.8	80.6	0.202
diameter ≥1	1	12.7	13.7	10.6		10.2	19.4	
Volume reduction (lobes)	0	46.5	49.5	40.4	0.291	56.2	41.7	0.345
	1	48.6	47.4	51.1		44.1	52.8	
	2	4.9	3.2	8.5		1.7	5.5	

*P*-values are for comparison between the respective groups. Bold values indicate statistically significant variables.

**FIGURE 1:**
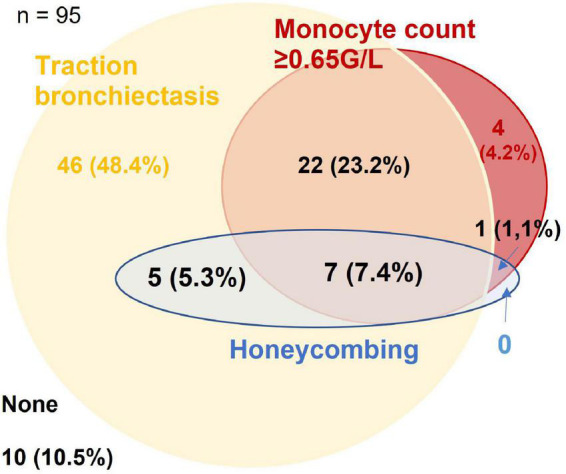
Venn-diagram for presence of traction bronchiectasis, honeycombing, and monocyte count ≥0.65 G/L in the derivation cohort. Figures are given as *n* (% of the cohort).

The optimum cut-off value for monocyte count was determined at ≥0.65 G/L (*p* = 0.008). Honeycombing, traction bronchiectasis and monocyte count ≥0.65 G/L were present in 13.7, 84.2, and 35.8% of patients, respectively, with overlaps as shown in Figure 1. A total of 7.4% of patients had evidence of all three domains, 10.5% had none.

**FIGURE 2:**
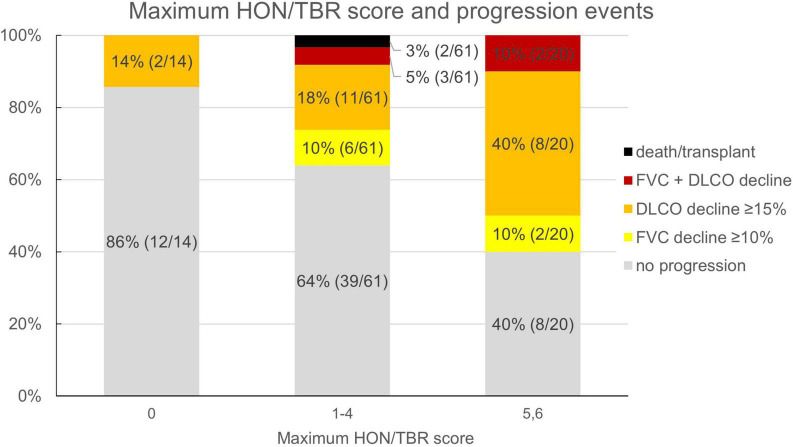
Honeycombing/traction bronchiectasis score and fraction (number) of progression events. HON/TBR, honeycombing/traction bronchiectasis; FVC, forced vital capacity; DLCO, diffusion capacity for carbon monoxide.

**FIGURE 3:**
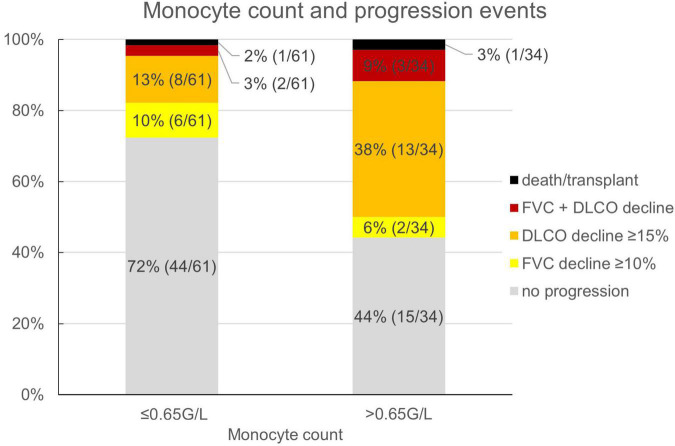
Monocyte count and fraction (number) of progression events. FVC, forced vital capacity; DLCO, diffusion capacity for carbon monoxide.

Reflecting the relatively low number of patients presenting with honeycombing, we implemented a combined score of the maximum honeycombing or traction bronchiectasis (HON/TBR) extent. The optimum cut-off values for limited and abundant extent of the leading HRCT variables honeycombing and traction bronchiectasis were determined at 0, 1–4, and 5–6 lung fields, respectively. The combined variable could also be shown to have a statistically significant interaction with progression at 1 year (*p* = 0.023). The relationship of HON/TBR extent as well as of monocyte count with number and fraction of progression events is shown in Figures 2,3.

Both variables, together with other known prognostic biomarkers and variables showing marked differences in initial analyses, were included in a regression model as shown in Table 4.

**TABLE 4:** Uni- and multivariate models for progression at 1 year and scoring of significant variables.

Variable	Univariate		Multivariate		Score points
	OR (95% CI)	*P*-value	OR (95% CI)	*P*-value	
Age ≥70 vs. <70 years	2.29 (0.98–5.35)	0.055			–
Sex (female vs. male)	0.68 (0.29–1.61)	0.191			–
Traction bronchiectasis/honeycombing 1–4 vs. 0	3.39 (0.69–16.5)	0.132	3.38 (0.67–17.3)	0.142	1
Traction bronchiectasis/honeycombing 5–6 vs. 0	**9.00 (1.57**–**51.46)**	**0.014**	**8.54 (1.43**–**51.2)**	**0.019**	**2**
Reticular lung abnormalities 5–6 vs. 0–4	2.48 (0.97–6.37)	0.059			–
Blood monocyte count ≥0.65 vs. <0.65 G/L	**3.28 (1.36**–**7.89)**	**0.008**	**3.16 (1.27**–**7.88)**	**0.014**	**1**
Blood lymphocyte count ≥1.6 vs. <1.6 G/L	1.02 (0.44–2.34)	0.971			–
Forced vital capacity (L)	0.98 (0.65–1.49)	0.933			–
Diffusion capacity for carbon monoxide [mmol/(min × kPa)]	0.94 (0.72–1.22)	0.632			–

Reticular lung abnormalities score of 0 was only present in one patient, thus the scores were merged to 0–4. OR, odds ratio; CI, confidence interval. Bold values indicate statistically significant variables.

**FIGURE 4:**
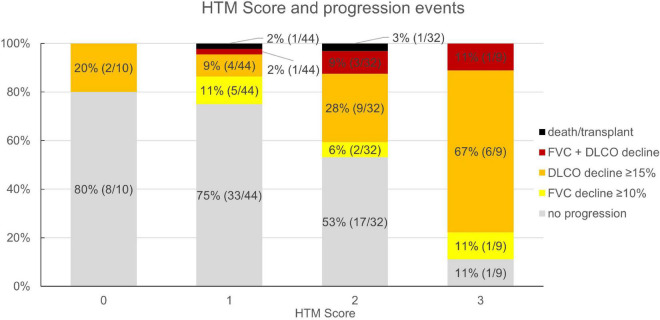
Honeycombing, traction bronchiectasis, and monocyte score and fraction (number) of progression events in the derivation cohort (*n* = 95). HTM, honeycombing, traction bronchiectasis, and monocyte; FVC, forced vital capacity; DLCO, diffusion capacity for carbon monoxide.

Based on the multivariate analysis results, a clinical score to assess progression-risk was derived by dividing the respective odds ratios by four and then rounding to even numbers. The score was subsequently referred to as the Honeycombing, Traction bronchiectasis and Monocyte (HTM)-score. As shown in Table 4, 1 point was counted for evidence of limited HON/TBR (scores 1–4) and for monocyte count ≥0.65 G/L, 2 points were counted for abundant HON/TBR (scores 5–6). This led to a maximum score of three for patients with abundant HON/TBR and elevated monocytes. In the derivation cohort, scoring resulted in progression rates of 20% in patients with 0 points (2/10), 25% for 1 point (11/44), 46.9% for 2 points (15/32), and 88.9% for 3 points (8/9) as shown in Figure 4, together with the number and fraction of progression events.

In the validation cohort, similar results could be shown: In the 45 of 47 evaluable patients (two patients had no blood monocyte count available), patients with a score of 0 progressed in 0% (*n* = 0/3), those with 1 in 23.8% (*n* = 5/21), with 2 in 53.9% (*n* = 7/13), and with 3 in 62.5% (*n* = 5/8). The ROC curve had an area under the curve of 71.7% as shown in Figure 4. Under the assumption of a score ≥2 as cut-off for progression, the score model showed a sensitivity of 70.6% and a specificity of 67.9%.

The same analyses were also attempted using the cut-off values for progressive pulmonary fibrosis (PPF) recently suggested by the novel ATS/ERS/JRS/ALAT guidelines (12), using absolute instead of relative decline and lower cut-offs of a 5% FVC and 10% DLCO decline to denote progression. A slightly higher portion of patients (two more) had progressive disease using this classification in the whole patient cohort. Forty-three (30%) had progression in both models, 13 (9%) had progression only using absolute, 11 (8%) only using relative lung function decline, while 75 (53%) did not progress in both models. Applying these cut-off values to the derivation cohort analogously to the previously described approach, no variable showed statistical significance.

## Discussion

Our findings from this retrospective, registry-based score evaluation and validation study involving patients with fibrotic ILD suggest that disease progression within 1 year was associated with the extent of honeycombing and/or traction bronchiectasis and peripheral blood monocyte count. We propose the HTM score as a prognostic tool for assessing progression-risk in fibrotic ILD patients, regardless of their underlying diagnosis or treatment.

**FIGURE 5:**
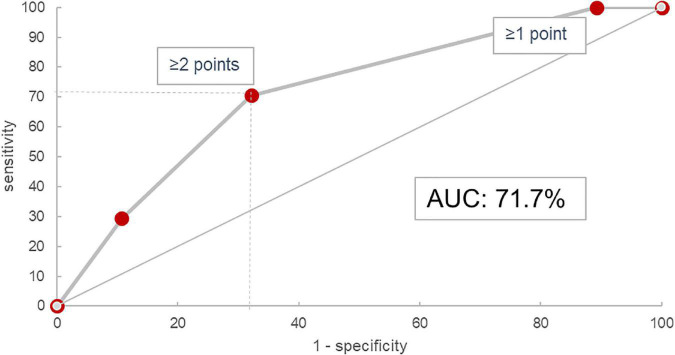
Receiver operating characteristic curve for the HTM score in the validation cohort. AUC, area under the curve.

Our findings integrate well into the existing knowledge on prognostic biomarker scores already described in various ILD, the most commonly used being the GAP-score originally developed for IPF patients and the staging algorithm by Goh et al. for SSC-ILD (22, 27). These indicate higher risk for male sex, older age, larger disease extent, and more advanced lung function impairment, respectively, however in very distinct cohorts: IPF patients are known to be predominantly male and usually of an advanced age (12, 37, 38), while SSC-ILD patients are more likely female, younger and more frequently show active lung inflammation (39–41). In our presented cohort, a larger variety of fibrotic ILD patients were evaluated together, comprising patients with ILD associated with autoimmune diseases or autoimmune features, idiopathic NSIP, chronic HP, and IPF. Apart from IPF, which expectedly had the highest progression rate (57%), all other major diagnostic subgroups consistently showed progression rates between 30 and 40% (Supplementary Table 2), which integrates well into existing evidence (12, 14). Importantly, results of sensitivity and specificity analyses as shown in the ROC curve in Figure 5 were comparable with those of established prognostic scores such as GAP and the composite physiologic index (CPI) used for assessment of mortality risk (22, 42), or the SPO_2_ and ARthritis (SPAR) model used for prognosis of progression in SSC-ILD (43).

Still, our proposed HTM-score with an AUC of 71.7% is certainly not a perfect prognostic tool. Alone, it should neither be used for therapeutic decisions, nor does it alleviate the expert physician’s responsibility to individually assess and follow every ILD patient thoroughly. However, there is rapidly increasing evidence that fibrotic ILD progression is paralleled by high mortality and that anti-fibrotic therapies should be established as soon as possible in such cases. We know from between-trial comparisons of placebo-groups in various trials on nintedanib that progression rate in non-IPF ILD like SSC-ILD may be lower as compared to IPF, but the net therapeutic effect of anti-fibrotics on disease progression itself seems comparable in different fibrotic ILD entities (8–11, 44, 45). Nevertheless, at the moment most treatment guidelines and expert opinions regarding non-IPF ILD such as ILD associated with autoimmune diseases or HP suggest anti-inflammatory drugs or observation as first-line option (13, 14, 46–48), while anti-fibrotic treatment with nintedanib is only recommended upon evidence of significant fibrotic disease progression (12). Still, fibrotic ILD progression can occur early and is usually irreversible. In contrast, reported response rates to anti-inflammatory therapies in fibrotic ILD are only modest and furthermore, such treatment can also result in increased morbidity and mortality in some patients (49). A considerable fraction of patients would undoubtedly benefit from earlier initiation of anti-fibrotic therapy, either alone or in combination with anti-inflammatory drugs. Our proposed score allows for a reasonably accurate estimation of progression-risk within the first year, based on widely available and easy to assess routine biomarkers. It could thus facilitate early initiation of anti-fibrotic treatment by prompting either more aggressive therapy earlier in the course of disease or at least closer monitoring for progression in patients identified to be at high risk.

We are aware that only recently, lower FVC and DLCO cut-off values for disease progression in PPF have been suggested by the ATS/ERS/JRS/ALAT guidelines (12), and our model could not be reenacted using these. However, one must keep in mind that these novel lung function progression criteria are intended to be applied together with clinical and radiological measures of disease progression that were not available in follow-up of our patients. Thus, our applied thresholds for progression necessarily needed to be higher to differ between clinically significant deterioration and physiological variation with a reasonable sensitivity and specificity. In line with that, Pugashetti et al. recently showed that a previous decline in FVC of ≥10% was the best biomarker for 5-year transplant-free survival in non-IPF ILD patients (50).

In our cohort, DLCO decline was the most frequent indicator of disease progression. However, DLCO had not been widely adopted as biomarker of disease progression in ILD until recently (12), due to its known methodologically determined variability and a variety of confounding factors like emphysema or pulmonary hypertension (12, 30, 51–53). We have analyzed progression-risk in association with presence and extent of emphysema as well as with pulmonary artery to aorta diameter as a surrogate for pulmonary hypertension and did not find statistically significant or clinically meaningful interactions as shown in Table 1. Also, despite its limitations, assessment of DLCO decline was included in the 2022 PPF criteria, due to its well established association with mortality in various ILD (12, 54, 55). A threshold of 15% relative decline in DLCO has repeatedly been used to denominate progression in various ILD studies (11, 43, 53), however this was now replaced by a threshold of 10% absolute decline in the recent guidelines (12). Concerning our statistical methods, the use of interpolation to assess the course of lung function variables at 1 year may require further discussion. However, an exploratory analysis excluding all patients with missing PFT at 1 year ± 2 months from the validation cohort (*n* = 17; 36%) showed a nearly equal AUC of 71.6% in the ROC analysis as explicated in Supplementary Figure 1 and Supplementary Table 3.

Another obvious limitation of this study is the absence of exact quantification of radiological changes. In our scores, only presence or absence of various radiological changes was assessed in the defined lung fields, but not the exact quantity of these changes within these fields. Thus, also the determined cut-off values to denote limited and abundant extent bear some uncertainty and may reduce comparability with other studies. Rather than exact quantification, our radiological evaluation approach reflects a fast and “eyeballing” evaluation of either absence, limited presence, or abundance of defined HRCT abnormalities. Therefore, it can be performed rapidly and requires neither costly software, nor a specialist radiologist. It may also be advantageous that the occasionally difficult differentiation between honeycombing and traction bronchiectasis is not necessary here (56). Nevertheless, an exact quantification of radiological abnormalities would be feasible using computer-based quantification algorithms, but these are not yet available to the wider clinical practice. In any case, our results of honeycombing and traction bronchiectasis being the main prognostic imaging biomarkers towards disease progression are well in line with studies using both visual scoring approaches and computer-based quantification systems (19, 21, 57).

Monocyte count has been repeatedly reported as significant prognostic biomarker for disease progression in various ILD (24, 58, 59), however it may be altered by extrapulmonary factors such as infections or medication(60–62). On the other hand however, routine blood cell counts are widely available and cheap to assess. Associations of immunomodulatory drugs with monocyte counts have been evaluated in smaller studies and indicated no or only small influence of such therapies (63, 64). Anti-fibrotic treatment on the other hand may decrease peripheral blood monocyte counts (59). In our cohort however, the majority of patients received ILD-specific treatment only after initial evaluation and inclusion into the ILD registry, so that such treatment effects are unlikely to have influenced the presented outcomes.

Pending further validation, the HTM-score can only be interpreted in the context of the underlying patient collective, which included a broad spectrum of different fibrotic ILD consecutively discussed by an experienced ILD-board in a university tertiary referral hospital. This may have led to the inclusion of rather complex cases, especially with an emphasis on ILD in rheumatological conditions, likely at the cost of more overt cases like IPF or sarcoidosis. Patients were included into this study regardless of their consecutive therapy which may have influenced the individual disease course over the first year. Only a minority of patients received anti-fibrotic therapy, which may be due to the more restrictive prescription regulations at the time of evaluation. On the other hand, a wide variety of anti-inflammatory therapies were applied, most commonly corticosteroids, and non-biological disease modifying drugs, at different doses and durations. Therefore, our classification of “anti-inflammatory therapy” constitutes only a minimum consensus for a very heterogeneous variable, which was necessary to enable any statistical analysis. Using random assignment to a derivation and a validation cohort, we sought to minimize temporal variability within the cohort. In addition, it appears unlikely that one diagnostic or therapeutic subgroup could have biased our results: Diagnosis categories (Supplementary Table 2) and treatment characteristics (Table 1) showed no significant interaction with disease progression and treatment modalities were well balanced between diagnostic subgroups, with the exception of a higher usage of anti-inflammatory medication in CTD-ILD (Supplementary Figure 2).

We conclude that our proposed HTM score was effective for prognosis of progression within the first year in a cohort of fibrotic ILD patients. This could enable earlier detection of progressive fibrosis and aid timely initiation of adequate therapy. Our results reflect the current knowledge of prognostic biomarkers in fibrotic ILD, and they could be reenacted in a randomly assigned validation cohort. Still, these findings warrant further validation in larger cohorts and using enhanced imaging modalities like computer-based HRCT quantification tools.

## Data availability statement

As mandated by the ethics committee, publication or dissemination of any possibly identifiable patient data from the present registry is prohibited. The dataset used for the present analyses contains very detailed and thus possibly identifiable patient data. Therefore, publication of the full database is not possible. However, upon reasonable request to the authors and if permitted by the ethics committee in an amendment to the study protocol, anonymized data can under certain circumstances be shared.

## Ethics statement

The studies involving human participants were reviewed and approved by the Ethics Committee of the Medical Faculty of Linz. The patients/participants provided their written informed consent to participate in this study.

## Author contributions

All authors listed have made a substantial, direct, and intellectual contribution to the work, and approved it for publication.

## Footnotes

1
https://www.R-project.org
