# Supplementary material for: Clinical, imaging, and blood biomarkers to assess 1-year progression risk in fibrotic interstitial lung diseases—Development and validation of the honeycombing, traction bronchiectasis, and monocyte (HTM)-score
Source: Front Med (Lausanne). 2022 Nov 16;9:1043720. doi: 10.3389/fmed.2022.1043720 (PMC9709148; doi:10.3389/fmed.2022.1043720)
Supplement: Supplementary file 1 [file Data_Sheet_1.docx]

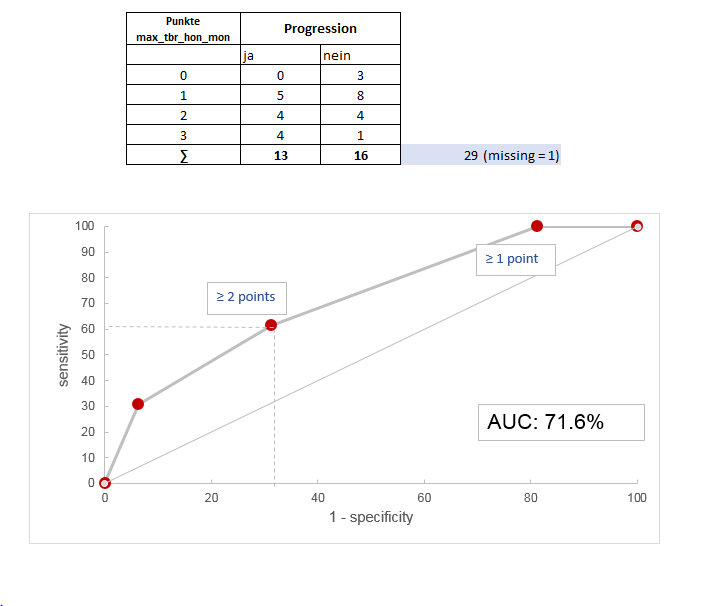
Supplementary figure 1. Receiver operating characteristic curve for the HTM score in the validation cohort excluding patients with missing lung function tests at one year +/- 2 months (n=30). AUC=area under the curve, HTM=honeycombing, traction bronchiectasis and monocyte
