# Supplementary material for: Clinical, imaging, and blood biomarkers to assess 1-year progression risk in fibrotic interstitial lung diseases—Development and validation of the honeycombing, traction bronchiectasis, and monocyte (HTM)-score
Source: Front Med (Lausanne). 2022 Nov 16;9:1043720. doi: 10.3389/fmed.2022.1043720 (PMC9709148; doi:10.3389/fmed.2022.1043720)
Supplement: Supplementary file 2 [file Data_Sheet_2.docx]

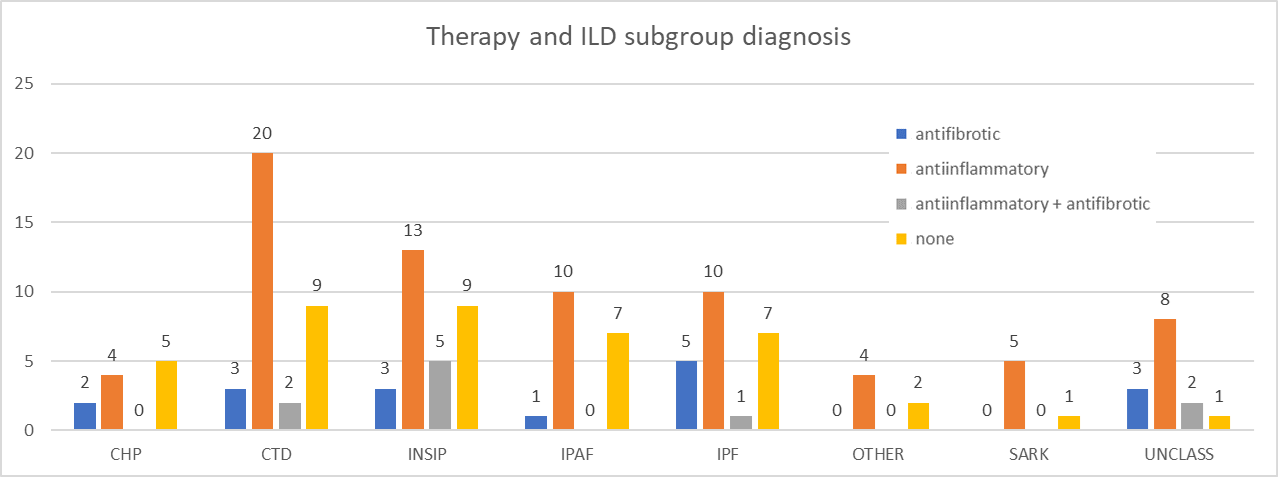


Supplementary figure 2. Main ILD diagnostic subgroups and ILD-specific therapy in the first year. The group of “other” ILD refers to the categorization shown in supplementary table 1. ILD=interstitial lung disease, CTD=connective tissue disease, INSIP=idiopathic non-specific interstitial pneumonia, IPAF=interstitial pneumonia with autoimmune features, IPF=idiopathic pulmonary fibrosis, SARK=sarcoidosis, UNCLASS=unclassifiable ILD
