# Supplementary material for: Clinical, imaging, and blood biomarkers to assess 1-year progression risk in fibrotic interstitial lung diseases—Development and validation of the honeycombing, traction bronchiectasis, and monocyte (HTM)-score
Source: Front Med (Lausanne). 2022 Nov 16;9:1043720. doi: 10.3389/fmed.2022.1043720 (PMC9709148; doi:10.3389/fmed.2022.1043720)
Supplement: Supplementary file 3 [file Table_1.docx]

| ILD main diagnosis | ILD subgroup diagnosis (n) | n (%) |
| --- | --- | --- |
| Chronic hypersensitivity pneumonitis |  | 11 (8) |
| Connective tissue disease-associated ILD |  | 34 (24) |
|  | Rheumatoid arthritis (10) |  |
|  | Systemic Sclerosis (10) |  |
|  | Antisynthetase syndrome (2) |  |
|  | Autoimmune myositis (3) |  |
|  | ANCA-associated Vasculitis (2) |  |
|  | Systemic lupus erythematodes (2) |  |
|  | Sjögren’s syndrome (2) |  |
|  | Goodpasture Syndrome (1) |  |
|  | Other autoimmune disorder related to ILD (2) |  |
| Idiopathic NSIP |  | 30 (21) |
| IPAF |  | 18 (13) |
| IPF |  | 23 (16) |
| Other ILD |  | 6 (4) |
|  | Cryptogenic organizing pneumonia (3) |  |
|  | Drug-associated (1) |  |
|  | Eosinophilic pneumonia (1) |  |
|  | Post-COVID-19-ILD (1) |  |
| Sarcoidosis |  | 6 (4) |
| Unclassifyable ILD |  | 14 (10) |
|  |  |  |

Supplementary table 1. Distribution of ILD-board diagnoses including subdiagnoses in all patients. ILD=interstitial lung disease, ANCA=anti-neutrophil cytoplasmatic antibody, NSIP=non-specific interstitial pneumonia, IPAF=interstitial pneumonia with autoimmune features, IPF=idiopathic pulmonary fibrosis, COVID-19=coronavirus disease 2019
