# Supplementary material for: Clinical, imaging, and blood biomarkers to assess 1-year progression risk in fibrotic interstitial lung diseases—Development and validation of the honeycombing, traction bronchiectasis, and monocyte (HTM)-score
Source: Front Med (Lausanne). 2022 Nov 16;9:1043720. doi: 10.3389/fmed.2022.1043720 (PMC9709148; doi:10.3389/fmed.2022.1043720)
Supplement: Supplementary file 4 [file Table_2.docx]

| ILD main diagnosis | n | No progression at one year | Progression at one year | % with progression at one year |
| --- | --- | --- | --- | --- |
| Chronic hypersensitivity pneumonitis | 11 | 7 | 4 | 36 |
| Connective tissue disease-associated ILD | 34 | 23 | 11 | 32 |
| Idiopathic NSIP | 30 | 19 | 11 | 37 |
| IPAF | 18 | 11 | 7 | 39 |
| IPF | 23 | 10 | 13 | 57 |
| Other ILD* | 26 | 19 | 7 | 27 |
|  |  | p=0.296 | |  |

Supplementary table 2. Main diagnostic subgroups and one year progression rate. *The group of “other ILD” summarizes the remaining groups presented in supplementary table 1. The p value refers to progression rates in the relative subgroups and was calculated using a Chi-square test. ILD=interstitial lung disease, NSIP=non-specific interstitial pneumonia, IPAF=interstitial pneumonia with autoimmune features, IPF=idiopathic pulmonary fibrosis
