# Supplementary material for: Clinical, imaging, and blood biomarkers to assess 1-year progression risk in fibrotic interstitial lung diseases—Development and validation of the honeycombing, traction bronchiectasis, and monocyte (HTM)-score
Source: Front Med (Lausanne). 2022 Nov 16;9:1043720. doi: 10.3389/fmed.2022.1043720 (PMC9709148; doi:10.3389/fmed.2022.1043720)
Supplement: Supplementary file 5 [file Table_3.docx]

| HTM score points | Progression at one year | No progression at one year | ∑ |
| --- | --- | --- | --- |
| 0 | 0 | 3 | 3 |
| 1 | 5 | 8 | 13 |
| 2 | 4 | 4 | 8 |
| 3 | 4 | 1 | 5 |
| ∑ | 13 | 16 | 29 |
|  |  | Missing values | 1 |

Supplementary table 3. Progression according to the HTM score in the validation cohort excluding patients with missing lung function tests at one year +/- 2 months (n=30). HTM=honeycombing, traction bronchiectasis, monocytes
